# Explaining Vegetable Consumption among Young Adults: An Application of the Theory of Planned Behaviour

**DOI:** 10.3390/nu7095357

**Published:** 2015-09-10

**Authors:** Davide Menozzi, Giovanni Sogari, Cristina Mora

**Affiliations:** Department of Food Science, University of Parma, via Kennedy 6, 43125 Parma, Italy; E-Mails: giovanni.sogari@unipr.it (G.S.); cristina.mora@unipr.it (C.M.)

**Keywords:** vegetables consumption, theory of planned behaviour (TPB), structural equation model (SEM), intention, background factors

## Abstract

Although fruit and vegetable consumption is highly recommended for a healthy and balanced daily diet, several European countries do not meet these recommendations. In Italy, only 45% of young people are consuming at least one portion of vegetables per day. Therefore, this paper aims to understand the main determinants of vegetables consumption among young adults to suggest possible intervention strategies. A cross-sectional study was conducted on a samples of Italian students (*n* = 751), using the theory of planned behaviour (TPB) as a conceptual framework. A structural equation model (SEM) was developed to test the TPB predictors for vegetable consumption, and the role of background factors (socio-demographic and personal characteristics) in improving the TPB model’s explaining power. Overall, 81% and 68%, respectively, of intentions and behaviour variance is explained by the TPB model. Socio-demographic and personal characteristics were found to influence intentions and behaviour indirectly by their effects on the theory’s more proximal determinants. Interventions should be targeted to improve perceived behavioural control (PBC), attitudes and subjective norms that significantly affect intentions. Tailored interventions for male students, enrolled in courses other than food science, and doing less physical activity may have a larger effect on behavioural change.

## 1. Introduction

Fruit and vegetables (F&V) are important elements for a healthy, balanced daily diet, bringing us vitamins, minerals, fibre, some energy, and other minor components such as phytochemicals which are potentially beneficial for our health. Proper nutrition and high intakes of fruit and vegetables assist in preventing a number of chronic diseases, including hypertension, cardiovascular disease, type 2 diabetes, certain cancers and musculoskeletal disorders [1,2,3].

The World Health Organization (WHO) and the Food and Agriculture Organization of the United Nations (FAO) recommend the intake of a minimum of 400 g of F&V (excluding potatoes and other starchy tubers) required by an individual per day for the prevention of chronic diseases and alleviation of several micronutrient deficiencies [1]. Several countries have translated this target into the “Eat five fruit or vegetables servings a day” message (in short: “5 A Day”). However, not all the countries meet these requirements; even in the EU several member states fail to meet this F&V intake [2]. Household data presented by the European Food Information Council (EUFIC) show that total F&V consumption ranged from 577 g/day in Poland to 196 g/day in Iceland, and vegetable consumption varied from a minimum of 109 g/day in Norway to a maximum of 284 g/day in Cyprus. Given these large discrepancies among European countries, the European Commission is monitoring the consumption of F&V as one way to offset a worsening trend of poor diets in Europe [3].

In Italy, which reported in 2000 the second F&V intake in Europe (approximately 450 g/day) and the highest consumption of processed vegetables (56 g/day) [2], the picture changed during the economic crisis with a drop in per capita consumption which affected more the consumption of fruit (−15% compared to 2000) than vegetables (−6%). Then, F&V consumption remained relatively stable from 2008 to 2013, so that in 2014 the annual consumption of F&V was lower than the recommended intake [4]. Moreover, in Italy, as well as in most countries, older people (aged 65 and over) more commonly eat vegetables daily, whilst consumption decreases among young people aged 15–24 years: only 45% of the population between 20 and 24 years consumes at least one portion of vegetables per day [4]. Therefore, explaining and understanding important factors affecting vegetable consumption among young adults in Italy is a necessary step in the development of an effective educational intervention to increase the intake of these essential food items. The present study aims to explain and test the main determinants of vegetables consumption among young adults in Italy using the theory of planned behaviour (TPB) [5] as a conceptual framework. The provided evidence may support the design of interventions to increase vegetable intake in this population.

## 2. Theoretical Framework

One of the more relevant theories to design evidence-based interventions is the TPB [5]. The TPB model postulates that attitude toward the behaviour, subjective norm (SN), and perception of behavioural control (PBC) lead to the formation of a behavioural intention, and that intention is assumed to be the immediate antecedent of behaviour. Intention captures the motivational factors that influence behaviour, e.g., to eat vegetables. As a general rule, the more favourable the attitude (*i.e.*, favourable or unfavourable evaluation of the behaviour) and subjective norm (*i.e.*, perceived social pressure), and the greater the perceived control (*i.e.*, perceived ability to perform the behaviour), the stronger should be the person’s intention to perform the behaviour in question. Given a sufficient degree of actual control over the behaviour, people are expected to carry out their intentions when the opportunity arises [6].

The TPB has been widely applied in predicting intentions and behaviour in many fields [7,8], including health-related behaviour [9] and F&V consumption [10]. However, the TPB studies focusing on vegetable consumption only are relatively few [10]. Although patterns of vegetable consumption among age groups and educational groups are similar to those for fruit [3], availability and psychosocial determinants of fruits and vegetables may be different [2,10]. Prior applications of the TPB in predicting vegetable consumption suggest that attitude, subjective norms and PBC explain 31% of the variance in intention and 10% of the variance in vegetable intake [10]. The intention to consume vegetables was mostly affected by individual’s beliefs about consequences and capabilities, as well as social influences. Vegetable intake, in turn, was predicted by behavioural intentions and beliefs about capability (*i.e.*, PBC). Therefore, consistent with the theory and the previous findings, in this study we suggest that:

H1: A favourable attitude would significantly predict intention to consume vegetables among young adults.

H2: Subjective norms would significantly predict intention to consume vegetables among young adults.

H3: PBC would significantly predict intention to consume vegetables among young adults.

H4: Behavioural intentions would significantly predict vegetables consumption among young adults.

H5: PBC would significantly predict vegetables consumption among young adults.

The TPB might not necessarily capture all of the predictors of more complex behaviour such as food choices [11]. Ajzen [12] has argued that, since the most detailed substantial information about the determinants of a given behaviour is contained in a person’s behavioural, normative and control beliefs, other background factors, such as socio-demographic characteristics or factors of a personal nature, are expected to influence intentions and behaviour indirectly by affecting the intention’s determinants attitude, subjective norm and PBC. Therefore, other variables, including socio-demographic characteristics of the sample, have extended the TPB improving its descriptive and predictive power in the literature. The socio-demographic variables included in the model were selected because they have been found to be significant moderators of vegetables intake in other studies, or because they were tested in systematic reviews [10,13]. Gender differences may moderate the vegetable intake: the Organisation for Economic Co-operation and Development (OECD) data show that more women reported eating vegetables daily than men [3], and it has been reported that females have more favourable attitudes and greater perceived behaviour control regarding F&V intake than males [14]. Similarly, preferences appear the strongest mediator of the gender difference in F&V intakes [15]. Age also seems to influence F&V consumption [2], where in European societies a decreasing intake of F&V is associated with increasing age of children and adolescents [13], whilst in adults the opposite seems true, *i.e.*, intake levels increase with age [16]. Other personal factors’ effects have also been studied; for instance, body mass index (BMI) is associated with vegetable consumption, where underweight people are usually less likely to meet the recommended targets [17]. Moreover, it was suggested that for many young adults’ living arrangements, in particular living away from the parents’ home, result in alterations to their food consumption habits in terms of fruits and vegetables consumption [18,19,20]. Papadaki *et al.* [18] found that students living away from the family home decreased their weekly consumption of fresh fruit, cooked and raw vegetables. A study performed in Southern Italy found contrasting results, since students living away from home were characterized by higher consumption of raw vegetables, whilst those living at home got higher quantities of cooked vegetables [20]. The same study supported the thesis that being responsible for food shopping and preparation (cooking meal) can lead to unhealthy dietary habits among university students living away from home. Similarly, it was found that living arrangement and shopping activity may affect fruit consumption [21]. Physical activity and F&V intake has been regarded to be related in several domains, especially when defining integrated public interventions to improve health behaviours [17,22,23,24]. In Italy, the place of origin may also contribute to explain the vegetable consumption, since dietary patterns of young population from southern regions are more traditionally adherent to the Mediterranean diet, therefore characterized by a higher consumption of vegetables and fruits, compared to the northern regions [25]. Background education, in terms of study area, has been found to influence F&V consumption behaviour [13,21]. Therefore, consistent with the theoretical framework and these findings, the model specification includes also other background factors, related to the socio-demographic and personal characteristics of respondents, in order to monitor their effect on intention determinants (*i.e.*, attitudes, subjective norms and PBC) and explore their potential in improving the explaining power of the TPB model.

H6: Gender, age, place of origin, living with family, BMI, food-related study, food shopping responsibility, cooking meals and physical activity would significantly affect attitudes.

H7: Gender, age, place of origin, living with family, BMI, food-related study, food shopping responsibility, cooking meals and physical activity would significantly affect subjective norms.

H8: Gender, age, place of origin, living with family, BMI, food-related study, food shopping responsibility, cooking meals and physical activity would significantly affect PBC.

**Figure 1:**
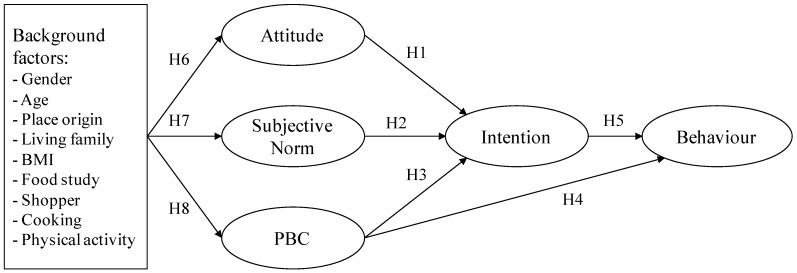
Specification of the theory of planned behaviour (TPB) model and tested hypothesis.

Therefore, this paper aims to test the TPB model predictors for vegetable consumption among young adults in Italy, and to explore the potential of background factors (socio-demographic and personal characteristics) to affect attitudes, subjective norms and PBC improving the explaining power of the TPB model, as displayed in Figure 1. Given the limited number of TPB studies focusing on vegetable consumption only, this paper could provide a framework for the definition of targeted interventions to counter decline in vegetable consumption among young adults in Italy.

## 3. Material and Methods

### 3.1. Data Collection and Sample

The cross-sectional sample consisted of 823 undergraduate students. Students were recruited from the University of Parma (Northern Italy) with advertisement on the university and faculties websites, and with announcements in classes and laboratories for participating to a general food consumption survey. Students were recruited in order to meet the University of Parma quota of areas of study (social, scientific and sanitary) and gender. Excluding the incomplete questionnaires, the final sample consisted of 751 students, 55% of which are females and 52% are usually living with parents at least five days per week (Table 1). The mean age was 22.1 ± 2.6 years old. Two third of the participants come from Northern Italy (67%), 5% from Central Italy, 27% from Southern Italy and only three students hailing from foreign countries.

**Table 1:** Characteristics of the study sample, percentage (%), mean and standard deviation (sd) (*n* = 751).

Sample Characteristics and Codes	
*Gender*	*%*
1 = Males	45.1
2 = Females	54.9
*Age*	mean (sd)
Age of participants	22.1 (2.6)
*Place of origin (Place origin)*	*%*
1 = Northern Italy	67.1
2 = Central Italy	5.2
3 = Southern Italy	27.3
4 = Other countries	0.4
*Living with family (Living family)*	*%*
1 = Never	32.5
2 = One or two days per week	8.9
3 = Three or four days per week	6.7
4 = Five or six days per week	4.7
5 = Everyday	47.3
*Body mass index (BMI)*	mean (sd)
BMI of participants (kg/m^2^)	22.2 (3.0)
*Students enrolled in food-related studies (Food study)*	*%*
0 = No	89.3
1= Yes	10.7
*Food shopping responsibility (Shopper)*	*%*
0 = No	55.3
1= Yes	44.7
*Cook meals (Cooking)*	*%*
0 = No	50.9
1= Yes	49.1
*Physical activity*	*%*
1 = Never	12.5
2 = Once per month	5.9
3 = Two or three times per month	12.6
4 = Once per week	17.3
5 = Two or three times per week	36.1
6 = From four to six times per week	12.3
7 = At least once per day	3.3

Note: the background factor name, if different than the sample class, is reported in parenthesis.

Participants have an average BMI, calculated based on self-reported height and weight, of 22.2 ± 3.0. BMI is normal ranged for 78% of students (BMI 18.5–24.9 kg/m^2^), while 15% of participants are overweight (BMI > 25 kg/m^2^) and 7% underweight (BMI < 18.5 kg/m^2^). Almost eleven percent of the participants are enrolled in University courses related to food (e.g., food science and technology, gastronomic science, *etc*.). Most participants (69%) reported practice of leisure-time physical activity at least once per week, while 12.5% were categorised into inactive (no reported physical activity). Only 45% of the respondents are the primary responsible for food shopping, while 49% usually prepare their own meals (Table 1). The sample has the same proportion of the population with respect to gender and studies. Data were collected during June and July 2013 with face-to-face interviews performed by three trained and experienced interviewers who submitted the TPB questionnaire with those who consented. All respondents participated in a lottery; five of them won a prize of 50 Euros.

### 3.2. Measures

The questionnaire items were defined taking into account Ajzen’s conceptual and methodological considerations in constructing a TPB questionnaire [5,6]. In particular, the Target, Action, Context, and Time (TACT) strategy was followed to define the behaviour (*i.e.*, “eating at least two servings of vegetables per day next week”). The TACT elements were defined considering the low level of vegetables consumption among young adults (only 45 percent eat at least one portion of vegetables per day), and an improvement to the average vegetable consumption of 1.4 servings per day of the Italian population [4]. Following the definition of the WHO, potatoes, sweet potatoes, cassava and other starchy roots were not classified as vegetables. A preliminary focus group and in-depth personal interviews with undergraduate university students were performed, as also recommended by Ajzen [6], to elicit salient expected outcomes and barriers connected to vegetable consumption.

The TPB items, as shown in Table 2, were scored on a 7-point Likert scale (in general, 1 = “totally disagree”, 7 = “totally agree”). Attitude toward the behaviour was assessed with four semantic differentials. Participants responded to the statement “Eating at least two servings of vegetables per day next week is: not pleasant/pleasant, not convenient/convenient, difficult/easy, not in line/in line with my food style”. Positive and negative endpoints were counterbalanced to avoid possible response set, and reversed for subsequent analysis. Four items assessed subjective norms: “My family/friends/my family doctor/the food industries and the retailers expect me to eat at least two servings of vegetables per day next week”. PBC was assessed with two items: “I think that eating at least two servings of vegetables per day next week is possible” and “Whether I eat at least two servings of vegetables per day next week is a decision that depends entirely on me”. Two items assessed behavioural intention: “I intend/I am sure to eat at least two servings of vegetables per day next week”. As noted by Ajzen [6], to obtain a reliable self-report measure of behaviour, it is desirable to use more than one question. Thus, two items were employed to measure behaviour, *i.e.*, vegetable consumption. Specifically, respondents were first asked to indicate from a list the number of servings of different vegetables (salad, pepper, cucumber, fennel, eggplant, carrot, tomato, *etc.*) consumed during the last 24 h. We also provided an example on the questionnaire explaining how to fill out this part. Then, we asked how many servings of vegetables have been eaten last week (ranging from 1 = less than three per week, to 6 = more than three per day). In this way, an estimate of the exact numerical report of servings consumed in the previous day and of the frequency of consumption was obtained. The internal consistency of the scales (Cronbach’s α), ranging from 0.49 (for behaviour) to 0.89 (for intention), suggests that the scales are reasonably homogenous (Table 2). The scales measuring behaviour generally have higher levels of internal consistency (*i.e.*, 0.60 or more), and 0.70 is often regarded as acceptable level for the reliability, although lower thresholds are also used in the literature [26]. However, since the reliability value obtained for behaviour (α = 0.49) is comparable to other studies [27], the application of two items, having the advantage of latent analyses, has been preferred.

### 3.3. SEM Analysis

A structural equation modelling (SEM) technique was employed on the data that were collected to test for the model identified in Figure 1. SEM may be considered as an extension of multiple regression, combining this statistical technique with (confirmatory) factor analysis (CFA). SEM allows for the specification of models structure with both latent and observed variables; latent variables, *i.e.*, abstract phenomena that cannot be directly measured by the researcher, have been analyzed using the CFA, often referred as the measurement model [28]. This is used when the researcher has some knowledge of the underlying latent variable structure or wishes to evaluate *a priori* hypotheses driven by theory. Relations between the latent variables identify the structural model. Using SEM it is possible to examine the influence of several variables on several other variables, according to a specified model. In SEM endogenous latent variables (*i.e.*, dependent variables) are influenced by the exogenous variables in the model either directly or indirectly, *i.e.*, mediated by other (endogenous) variables. Therefore, exogenous latent variables (*i.e.*, independent variables) “cause” fluctuations in the values of other endogenous latent variables in the model [28]. Thus, the whole TPB can be tested in relation to the dataset in one analysis [29].

The use of different goodness-of-fit indices is generally recommended to test how well the observed data fit the model. Model fit was assessed with chi-square (χ^2^), comparative fix index (CFI), the Tucker-Lewis Index (TLI), and root mean square error of approximation (RMSEA) with 90% confidence interval (CI). An adequate model fit is obtained when the CFI and TLI are >0.90 and the RMSEA <0.08, while a superior fit is obtained when the CFI and TLI are >0.95 and the RMSEA is <0.05. The coefficient of determination R-square was used to measure the explained variance of the endogenous variables. The models were estimated using Bayesian estimation procedure, suggested to analyse categorical data [28].

**Table 2:** Descriptive statistics, mean scores and standard deviations, Cronbach’s α, mean and standard deviation (sd).

Constructs Items	Mean (sd)
**Attitude** (α = 0.76)	
*Eating at least two servings of vegetables per day next week is*:	
- not pleasant/pleasant (reverse)	4.77 (1.62)
- not convenient/convenient	4.75 (1.59)
- difficult/easy (reverse)	5.14 (1.64)
- not in line/in line with my food style	5.12 (1.80)
**Subjective Norm** (α = 0.71)	
My family expects me to eat at least two servings of vegetables per day next week	4.50 (1.87)
My friends expect me to eat at least two servings of vegetables per day next week	2.81 (1.68)
My family doctor expects me to eat at least two servings of vegetables per day next week	5.02 (1.72)
The food industries and the retailers expect me to eat at least two servings of vegetables per day next week	4.12 (1.78)
**PBC** (α = 0.78)	
I think that eating at least two servings of vegetables per day next week is possible	5.30 (1.76)
Whether I eat at least two servings of vegetables per day next week is a decision that depends entirely on me	5.04 (1.79)
**Intention** (α = 0.89)	
I intend to eat at least two servings of vegetables per day next week	4.85 (1.83)
I am sure to eat at least two servings of vegetables per day next week	4.40 (2.02)
**Behaviour** (α = 0.49)	
Number of servings	2.98 (1.86)
Frequency of consumption ^a^	3.78 (1.28)

^a^ The frequency of consumption is measured by the following item: “How many servings of vegetables have been eaten last week: 1 = less than three, 2 = from three to five, 3 = one per day, 4 = two per day, 5 = three per day, and 6 = more than three per day”.

## 4. Results

### 4.1. Descriptive Analysis

Table 2 presents the descriptive statistics. Mean vegetable consumption was approximately three servings of vegetables per day (2.98 ± 1.86). When considering the frequency of consumption over the last week, on average the respondents reported a daily vegetable consumption of two servings (median class = 4).

The results showed a general positive attitude towards traceable eating at least two servings of vegetables per day next week. The respondents thought that vegetable consumption is easy (5.14 ± 1.64) and in line with their food style (5.12 ± 1.80), moderately pleasant (4.77 ± 1.62) and convenient (4.75 ± 1.59). According to the SN items, respondents perceive that especially family doctors (5.02 ± 1.72) and, to a lesser extent, family (4.50 ± 1.87) expect them to eat at least two servings of vegetables per day, while the food industries and retailers expectations are perceived as neutral (4.12 ± 1.78), and friends expectations are not perceived (2.81 ± 1.68). In other words, the participants generally perceived more the social pressure from family doctors and families than food industries, retailers and friends. Respondents generally perceived a high control over the behaviour; they believe that eating at least two servings of vegetables per day is possible (5.30 ± 1.76) and that it depends entirely on them (5.04 ± 1.79). Thus, self-efficacy with respect to vegetable consumption is generally positive. Respondents reported moderately positive intentions to eat at least two servings of vegetables per day next week (4.85 ± 1.83), are they are moderately sure to do that (4.40 ± 2.02).

### 4.2. Predicting Vegetable Consumption with the TPB

Goodness-of-fit statistics related to the TPB model revealed that the hypothesized model fits the data very well, as evidenced by the CFI of 0.981, TLI of 0.975 and RMSEA of 0.040 (90% CI = 0.031–0.048) (Table 3). Overall, 81% and 68%, respectively, of the intentions and behaviour variance is explained by the TPB model. These results are satisfactory considering that a meta-analysis found a random-effect *R*^2^ for the prediction of vegetable intake intention of 0.31 and behaviour of 0.13 [10]. However, the cross-sectional nature of this study may have inflated the relationship between psychosocial variables and behaviour.

**Table 3:** TPB model unstandardized coefficients (coeff), standard error (se), standardized coefficients (std), and *p*-values.

	***Endogenous Variables***										
	***Intention***					***Behaviour***					
***R^2^***	***0.81***					***0.68***					
***Determinants***	***coeff***	***se***		***std***	***p***	***coeff***		***se***		***std***	***p***
Attitude	0.47	0.07		0.29	***						
Subjective Norm	0.56	0.10		0.23	***						
Perceived Behavioural Control (PBC)	0.69	0.07		0.53	***	0.12		0.08		0.18	0.082
Intention						0.33		0.06		0.67	***
	***Covariances and Correlations***										
	***coeff***		***se***				***std***		***p***		
Attitude ↔ Subjective Norm	0.30		0.05				0.41		***		
PBC ↔ Subjective Norm	0.50		0.07				0.57		***		
PBC ↔ Attitude	0.93		0.09				0.69		***		

Model fit measures: χ^2^ (d*f*) = 145.911 (67), *p* = 0.000; comparative fix index (CFI) = 0.981; Tucker-Lewis Index (TLI) = 0.975; root mean square error of approximation (RMSEA) (90% confidence interval (CI)) = 0.040 (0.031–0.048). Signif. codes: *** = *p* < 0.001.

Attitudes, subjective norms and PBC are all significant predictors of intentions, therefore supporting respectively H1, H2 and H3. Table 3 shows that PBC is the main predictor of intentions (γ = 0.53, *p* < 0.001), followed by attitudes (γ = 0.29, *p* < 0.001) and subjective norms (γ = 0.23, *p* < 0.001). The variables attitude, PBC and subjective norms are all positively correlated, supporting the theoretical hypothesis of the TPB [5]. Behaviour is significantly affected by intentions (β = 0.67, *p* < 0.001), as predicted by H4. However, H5 is not confirmed since PBC affects behaviour only marginally (β = 0.18, *p* = 0.082).

Table 4 shows the results of the TPB-extended model obtained adding the background factors (gender, age, place origin, living family, BMI, food study, shopper, cooking and physical activity) to the model as influencing attitudes, subjective norms and PBC [12]. Goodness-of-fit statistics were also found to be still very good (CFI = 0.969, TLI = 0.959, RMSEA = 0.042, 90% CI = 0.036–0.049). Hypothesis H1, H2 and H3 are still confirmed since attitude, subjective norms and PBC are significant predictors of intentions. H4 and H5 are also confirmed since both intentions (β = 0.63, *p* < 0.001) and PBC (β = 0.22, *p* < 0.05) are significant predictors of behaviour. Overall, the explained variance of intentions (83%) and behaviour (69%) is slightly higher than the TPB model.

Attitudes, subjective norms and PBC are significantly influenced by a number of background factors, thus supporting H6, H7 and H8. In particular, attitude is influenced by gender (γ = 0.27, *p* < 0.001) indicating that females have a more positive attitude towards vegetables consumption than males. More favourable attitudes were found for those students being responsible for food shopping (γ = 0.32, *p* < 0.001), and for those doing regular physical activity (γ = 0.15, *p* < 0.001). Background education is also affecting attitudes, since students enrolled in food-related university courses have more positive attitudes towards eating vegetables (γ = 0.11, *p* < 0.01). Gender (γ = 0.29, *p* < 0.001), food study (γ = 0.18, *p* < 0.001), BMI (γ = 0.12, *p* < 0.01) and physical activity (γ = 0.11, *p* < 0.05) are significantly affecting subjective norms, indicating that female students, enrolled in food courses and doing regular physical activity with relatively higher BMI perceived more social pressure to eat at least two servings of vegetables per day next week. PBC is influenced by several socio-demographic variables like gender (γ = 0.26, *p* < 0.001), shopping responsibility (γ = 0.27, *p* < 0.001), food-related studies (γ = 0.16, *p* < 0.001), place of origin (γ = −0.17, *p* < 0.01), BMI (γ = 0.13, *p* < 0.01), physical activity (γ = 0.11, *p* < 0.01), and living with family (γ = 0.12, *p* < 0.05). This indicates that females from Northern Italian regions, with higher BMI, enrolled in food-related university courses, doing more regular physical activity, living more frequently with family and holding the primary shopping responsibility, perceived ease of eating vegetables.

Not surprisingly, several socio-demographic and personal characteristics are mutually correlated (Table 5). Females have lower BMI (φ = −0.35), hold more often the shopping (φ = 0.12) and cooking responsibility (φ = 0.14), and do less physical activity (φ = −0.25) than males. Older students are less likely to be enrolled in food courses (φ = −0.22) and are more often responsible for shopping (φ = 0.06, *p* < 0.05) than younger students. Students from Southern Italian regions generally live away from their family home (φ = −0.69), and therefore hold primary shopping (φ = 0.48) and cooking responsibilities (φ = 0.47) than those from Northern Italy. Students living more often with their family hold less shopping (φ = −0.65) and cooking responsibilities (φ = −0.59), and do more regular physical activity (φ = 0.08) than students living away from the family home. Finally, being responsible for food shopping is also correlated with food preparation responsibilities (φ = 0.69).

**Table 4:** TPB-extended model unstandardized coefficients (coeff), standard error (se), standardized coefficients (std), and *p*-values.

	*Endogenous Variables*																			
	*Intention*				*Behaviour*				*Attitudes*				*Subjective Norms*				*PBC*			
*R^2^*	*0.83*				*0.69*				*0.17*				*0.11*				*0.15*			
*Determinants*	*coeff*	*se*	*std*	*p*	*coeff*	*se*	*std*	*p*	*coeff*	*se*	*std*	*p*	*coeff*	*se*	*std*	*p*	*coeff*	*se*	*std*	*p*
Attitude	0.47	0.07	0.30	***																
Subjective Norm (SN)	0.58	0.10	0.24	***																
Perceived Behavioural Control (PBC)	0.68	0.07	0.52	***	0.17	0.08	0.22	0.032												
Intention					0.37	0.06	0.63	***												
Gender									0.58	0.10	0.27	***	0.42	0.08	0.29	***	0.69	0.11	0.26	***
Age									0.03	0.02	0.06	0.106	0.02	0.01	0.07	0.072	0.02	0.02	0.04	0.313
Place origin									−0.12	0.06	−0.10	0.060	−0.01	0.04	−0.02	0.765	−0.24	0.08	−0.17	0.001
Living family									0.07	0.04	0.11	0.082	0.01	0.03	0.01	0.824	0.08	0.04	0.12	0.038
Body mass index (BMI)									−0.01	0.02	−0.02	0.644	0.03	0.01	0.12	0.007	0.05	0.02	0.13	0.002
Food study									0.39	0.14	0.11	0.004	0.41	0.10	0.18	***	0.65	0.16	0.16	***
Shopper									0.69	0.13	0.32	***	0.11	0.09	0.08	0.200	0.70	0.15	0.27	***
Cooking									−0.06	0.12	−0.03	0.586	−0.02	0.08	−0.01	0.818	−0.16	0.14	−0.06	0.264
Physical activity									0.10	0.03	0.15	***	0.05	0.02	0.11	0.012	0.09	0.03	0.11	0.006

Model fit measures: χ^2^ (d*f*) = 416.204 (189), *p* = 0.000; comparative fix index (CFI) = 0.964; Tucker-Lewis Index (TLI) = 0.951; root mean square error of approximation (RMSEA) (90% CI) = 0.040 (0.035–0.045). Signif. codes: *** = *p* < 0.001.

**Table 5:** TPB-extended model: Covariances, standard errors (italic), correlations (bold).

	1	2	3	4	5	6	7	8	9	10	11	12
1. Attitude		0.22 *0.04* **0.35**	0.76 *0.08* **0.66**									
2. Subjective Norm (SN)			0.42 *0.06* **0.53**									
3. Perceived Behavioural Control (PBC)												
4. Gender					n.s.	n.s.	n.s.	−0.51 *0.05* −**0.35**	n.s.	0.03 *0.01* **0.12**	0.03 *0.01* **0.14**	−0.20 *0.03* −**0.25**
5. Age						n.s.	n.s.	n.s.	−0.18 *0.03* −**0.22**	0.07 *0.03* 0.06 *	n.s.	n.s.
6. Place origin							−1.22 *0.07* −**0.69**	n.s.	n.s.	0.22 *0.02* **0.48**	0.21 *0.02* **0.47**	n.s.
7. Living family								n.s.	n.s.	−0.58 *0.04* −**0.65**	−0.53 *0.04* −**0.59**	0.22 *0.06* **0.08**
8. Body mass index (BMI)									n.s.	n.s.	n.s.	n.s.
9. Food study										n.s.	n.s.	n.s.
10. Shopper											0.17 *0.01* **0.69**	n.s.
11. Cooking												n.s.
12. Physical activity												

Notes: All the covariances are significant at *p* < 0.001, * Shopper ↔ Age (*p* = 0.016); n.s.: not significant.

## 5. Discussion 

The results confirm the utility of the TPB in predicting the intention to consume at least two servings of vegetables per day among young adults; the TPB variables alone are able to explain 81% of intention while, when background factors (socio-demographic variables and personal characteristics) are included in the model, the explained variance increases up to 83%. Interestingly, intention and PBC account for 68% of behaviour (69% when adding background factors). Although cross-sectional designs are known to inflate the relationship between psychosocial variables and behaviour, a meta-analysis on fruit and vegetables intake determinants has shown a higher efficacy in prediction when using a longitudinal design [10]. The authors concluded that their findings provide moderate support for the higher efficacy in prediction when good psychometric quality instruments were used to assess psychosocial and behavioural measures. Intention is the main factor in predicting behaviour, indicating that vegetable consumption among young adults is strongly influenced by conscious deliberation. This result confirms other studies where intention was found to be a significant predictor of F&V intake [30,31,32]. A significant effect of PBC in predicting behaviour was found in the TPB-extended model. In other studies, perceived barriers to healthy eating were found to negatively affect vegetable intake [32,33], while cost and availability were found to be the major barriers to fruit and vegetable consumption among young adults in New Zealand [22]. In our case, those having higher self-efficacy for eating vegetables report higher vegetable consumption. PBC is also the main determinant of intentions, indicating that perceived ease or difficulty of performing the behaviour strongly influence the intention to perform the behaviour. Attitudes and subjective norms have been also found to positively affect intentions; concerns about health, taste preference and perceived benefit of healthy eating were also found as significantly affecting vegetable intake and intentions [33], whereas previous literature consistently found a significant relationship between subjective norm and intention to eat F&V [22,31,32]. Social influences, such as parents and family doctors, affect the intention of participants to eat at least two servings of vegetables per day. Other studies have found that flatmates had great influence by giving social support and acting as role models [22], as well as sharing groceries and cooking [19].

The TPB-extended model, although having only marginally improved the prediction of dependent variables, has demonstrated that background factors (socio-demographic characteristics and other personal factors) influence intentions and behaviour indirectly by their effects on attitude, subjective norms and PBC, as suggested by Ajzen [12]. Females reported greater PBC, attitudes and subjective norms regarding eating at least two servings of vegetables per day than males. Emanuel *et al.* [14] reported similar effects, with the only exception of subjective norms, since males reported greater perceived norms compared to females. Respondents doing regular physical activity reported more positive attitudes, a higher confidence and a stronger normative pressure regarding eating vegetables than those with lower level of physical activity. Background education, in particular being enrolled in food-related university courses, was also found to significantly affect PBC, subjective norms and attitudes. These students have demonstrated to have more consciousness about the outcomes related to vegetables consumption, more self-efficacy and higher perception of social pressure in promoting healthy eating. Therefore, we recommend controlling for these variables when doing future research in similar contexts. Being primary responsible for food shopping positively influences attitudes and perceived ability to eat vegetables. Several studies have argued that home food availability is correlated with both F&V intake during young adulthood [13,34]. This finding supports the thesis that assumption of primary responsibility for food shopping can lead to healthy dietary habits among university students, partially contradicting other studies [20]. Our results indicate that overweight and obese people have higher confidence and stronger normative pressure regarding eating vegetables than their normal weight counterparts. Other studies have demonstrated that BMI was significantly correlated to intention to eat five servings of F&V per day, and it was thus suggested to control for it in subsequent analyses [17,31]. Finally, the place of origin affects PBC indicating that students from Southern Italian regions perceived more barriers to vegetables consumption than students from the North. This result is quite surprising, since one would expect that young adults from Southern Italy were more traditionally adherent to the Mediterranean diet, and therefore consuming more vegetables. However, other studies have demonstrated that young adults from Southern Italian regions are shifting away from the Mediterranean dietary patterns [20], and our findings may support this argument.

According to the TPB, interventions should be directed at modifying salient beliefs in order to produce corresponding changes in attitudes, subjective norms and PBC which, in turn, may further influence intentions in the desired direction. However, as suggested by Ajzen [35], the intervention will be effective if individuals are capable of carrying out their formed intentions. This happens only if there is a strong link from intentions to behaviour. Thus, although it is reasonable to target an intervention at any one of the three major predictors in the TPB, it may be safer to consider their relative weights in the prediction of intentions and behaviour to target the intervention [35]. In the case presented, the respondents, which expressed a strong intention–behaviour link, can be targeted to improve, respectively, PBC, attitudes and subjective norms that significantly affect intentions, using a variety of media channels, including billboards and the internet [22]. To improve the perceived ability to eat vegetables it would be possible to provide instructions or “tips” on purchasing, storage and preparation of different varieties of vegetables, including information about places to get cheap vegetables and quick and easy recipes. Providing factual information on the material consequences of eating vegetables regularly, in particular short-term health implications, may improve consumers’ attitude. Finally, the perceived social pressure could be improved by providing more information about other same age peers behaviour related to vegetables consumption, or providing a setting in which social comparison can occur [30]. These interventions should be primarily targeted for male students, enrolled in university courses not directly related to food, and doing less physical activity. Indeed, a significant increase in attitudes, subjective norms and PBC in those categories may have a larger effect on behaviour change [11,35].

Nevertheless, as noticed by some authors [11,36], researchers need to exercise some caution in assuming that the TPB can provide a complete model of behaviour change. It may not always be the case that a change in the antecedents of behaviour (*i.e.*, intention, subjective norm, attitude, and PBC) will lead to change in vegetable consumption. The TPB has been severely criticised for its lack of suitability as a theory of behaviour change [36]. Ajzen has replied to these criticisms explaining that (i) the TPB is meant to help explain and predict people’s intentions and behaviour; and that (ii) the theory can serve as a useful framework for designing effective behaviour change interventions [37]. The first argument is also supported by the literature review performed by Guillaumie *et al.* [10], which suggests that the TPB performs well for the study of the determinants of fruit and vegetable intake and that the TPB seems an appropriate choice to predict intention and behaviour. The second, as also recognised by Ajzen, is more difficult to attain since the design of an “effective behaviour change intervention requires a great deal of preparation and formative research” [37] (p. 4). The same author noted that the TPB do not preclude addition of new predictors after careful deliberation and empirical exploration [5,12,37]. For instance, because of the habitual nature of the behaviour in question, people may find it difficult to act on their good intentions [21,38,39,40]. Future research is warranted to understand how habit strength may moderate vegetable consumption.

Some methodological limitations to the current study have to be addressed. First, although the use of a cross-sectional data and self-reported measures is common in several TPB studies [10], it presents conceptual problems in the causal ordering of the TPB and may have inflated the associations between TPB variables and behaviour [39]. Therefore, it should be noted that the psychosocial determinants probably are less strongly connected with actual vegetable consumption among young adults than what has been found in this study about their connection with self-reported intentions and behaviour. Moreover, strictly speaking, the indicators of the behaviour we have obtained refer to past behaviour (*i.e*., last week, last 24 h). However, the Italian Institute for Studies, Research and Information on the Agricultural Food Market [41] shows limited variation in vegetable consumption from 2008 to 2013 (annual variation −0.20%) and little variation during the summer months, when the data were collected. Therefore, we feel fairly confident that our measure of vegetable consumption would not have changed much if it was assessed longitudinally. Future research, however, should focus on longitudinal data to make stronger assertions of causality between the TPB variables and vegetable consumption. Second, the sample represents a highly educated segment of consumers; since highly educated persons tend to eat vegetables more often [3], a generalization to the general Italian young adult population is difficult. Third, we have violated the principle of compatibility [6] since the self-report measure of behaviour used in this study required participants to estimate vegetable consumption retrospectively, whilst the TPB variables were designed to assess a prospective behaviour (*i.e.*, “eating at least two servings of vegetables per day next week”). Despite these limitations, our study is one of the first to explore the determinants of vegetable consumption in a context of declining fruit and vegetable intake. These outcomes will assist the development of tailored interventions to promote vegetable consumptions in a target population of the EU policies. We acknowledge that this research would have been more effective if we had designed the intervention and measured its effect in changing the young adults’ behaviour. Therefore, further research efforts should use longitudinal data to investigate how theory-based interventions would be effective in changing young adults’ behaviour.

## 6. Conclusions

In conclusion, the current study provides further support for the TPB in testing the main determinants of vegetables consumption among young adults in Italy. It also explores the potential of socio-demographic and personal characteristics as background factors in affecting attitudes, subjective norms and PBC. Attitudes, subjective norms and PBC are all significant predictors of intentions, while behaviour is significantly affected by intentions. This suggests that efforts to increase intentions through targeting attitudes, subjective norms and PBC may have a knock on effect in increasing vegetable consumption. Gender, food-related study and physical activity are the background factors that significantly affect attitudes, subjective norm and PBC. Given the limited number of TPB studies focusing on vegetable consumption only, this paper provides a framework for the definition of targeted interventions to counter decline in vegetable consumption among young adults in Italy. 
